# Round Ligament Endometriosis Mimicking Chronic Postoperative Inguinal Pain Following Robotic Inguinal Hernia Repair: A Case Report and Brief Literature Review

**DOI:** 10.7759/cureus.112592

**Published:** 2026-07-13

**Authors:** Zac W Riggenbach, Jonathan Lutgens, Brooklyn Williams, Abigail Kelly, Jason Bingham

**Affiliations:** 1 General Surgery, Madigan Army Medical Center, Tacoma, USA

**Keywords:** chronic postoperative inguinal pain, groin pain, inguinal endometriosis, robotic hernia repair, round ligament endometriosis

## Abstract

Chronic postoperative inguinal pain (CPIP) affects an estimated 4%-21% of patients following inguinal hernia repair, with rates varying by technique, follow-up duration, and pain definition, and requires a systematic, multidisciplinary evaluation. Inguinal endometriosis is a rare entity, representing approximately 0.07% of all endometriosis cases, and is an uncommonly recognized cause of inguinal pain that may mimic CPIP. We present the case of a 37-year-old woman who developed new left-sided groin pain following an elective bilateral robotic transabdominal preperitoneal inguinal hernia repair. Initial workup, including physical examination and magnetic resonance imaging (MRI), was unremarkable, and conservative management with activity modification, analgesics, and a targeted nerve block provided no sustained relief. At 16 months postoperatively, the patient reported a palpable tender nodule near the left pubic symphysis with symptoms that had become clearly cyclical in nature. A repeat MRI timed to coincide with her menstrual cycle revealed soft tissue inflammation along the left round ligament near the pubic tubercle. Surgical excision of the round ligament with concurrent ilioinguinal neurectomy was performed, and histopathological analysis confirmed endometriosis. The patient experienced complete resolution of her cyclical pain postoperatively. A brief literature review of 21 published studies on inguinal and round ligament endometriosis was performed, which demonstrated that right-sided disease predominates, cyclical symptoms are common but not universal, and misdiagnosis is frequent. This case highlights the importance of considering inguinal endometriosis in the differential diagnosis of CPIP in women of reproductive age, particularly when symptoms are cyclical or localized outside the expected operative field. Additionally, this case underscores the potential diagnostic value of timing imaging studies to coincide with symptomatic windows to improve sensitivity for catamenial pathology.

## Introduction

Chronic postoperative inguinal pain (CPIP) represents a potentially challenging condition for both surgeons and patients. Recent advances in technique, including laparoscopic or robotic repairs, have been associated with improved long-term pain compared with traditional open repairs [1]. Despite these improvements, some cohort studies estimate CPIP between 4% and 21%, with between 2% and 4% of patients with debilitating chronic pain [1-3]. When present, CPIP requires a methodical, stepwise workup with a multidisciplinary team [4].

Inguinal endometriosis, however, is a rare entity representing only 0.07% of endometriosis cases [5]. It may be classified by the Niitsu classification system, with type I representing the most common presentation within a hernia sac or canal of Nuck lesion, type II lesions on the round ligament, and type III lesions in the subcutaneous tissue [6]. This case highlights a rare presentation of type II inguinal endometriosis developing after a minimally invasive inguinal hernia repair and subsequently mimicking CPIP.

## Case presentation

A 37-year-old otherwise healthy female with no past surgical or gynecologic history presented with a two- to three-year history of intermittent right groin bulge and vague pelvic pain. Her symptoms worsened with physical activity, particularly lifting, and the bulge was always reducible. She was initially evaluated by a gynecologist and underwent a transvaginal ultrasound due to her complaints of chronic pelvic pain. Unfortunately, her transvaginal ultrasound was unrevealing. A subsequent CT scan identified bilateral inguinal hernias, and she was referred to the general surgery clinic, where she was evaluated and scheduled for operative repair.

The patient underwent an elective bilateral robotic-assisted laparoscopic trans-abdominal pre-peritoneal (TAPP) inguinal hernia repair two months after her initial evaluation. Intraoperatively, bilateral indirect hernia defects were identified and both repaired. The hernia sacs were found densely adherent to the round ligaments bilaterally. To allow for complete reduction of the hernia sacs, both round ligaments were transected. Both hernia defects were repaired with a macroporous, medium weight, self-adhering 10 x 15 cm mesh, and the peritoneal flaps were closed. The remainder of the operation was unremarkable.

At her one-month postoperative visit, the patient reported new-onset left groin pain near the pubic symphysis. Notably, this pain was unique from her preoperative symptoms, which had been isolated to the right side. On examination, there was no evidence of hernia recurrence. She was otherwise progressing well postoperatively, so the patient trialed conservative management, including activity modification.

**Figure 1:**
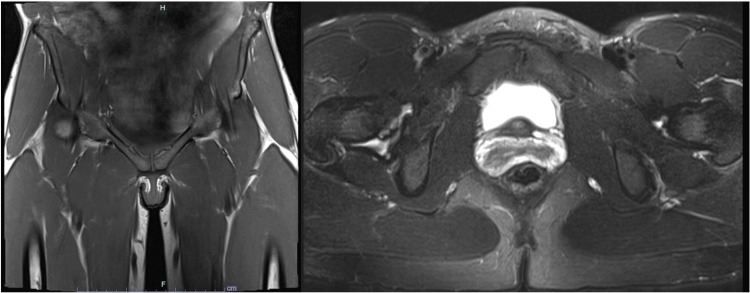
MRI from eight-month follow-up This figure shows MRI results of the patient, performed approximately eight months after the initial robotic-assisted inguinal hernia repair. Note that there appears to be no evidence of inflammation near the round ligament insertion and the mons pubis, or of hernia recurrence. The patient was not currently experiencing symptoms at the time of this study. MRI, magnetic resonance imaging.

Eight months postoperatively, the patient returned with persistent left-sided pain again localized to the area around the pubic symphysis and exacerbated by activity. Examination revealed point tenderness but no palpable hernia. A magnetic resonance imaging (MRI) was performed and showed no evidence of hernia recurrence or other significant abnormalities, as shown in Figure 1. She was advised to continue rest and oral analgesics. Additionally, she was referred to a chronic pain specialist for targeted nerve therapy. She underwent a single injection targeting the ilioinguinal nerve, which provided transient relief for only a few minutes.

At 16 months postoperatively, the patient returned to the general surgery clinic with continued pain and new symptoms. She reported a palpable nodule just to the left of the pubic symphysis in the area of maximal discomfort. She also noted that her symptoms had become more clearly associated with her menstrual cycle over the previous few months. At the time of evaluation, she was near the onset of her menstrual period, and examination revealed a distinct, tender nodule with significant pain on palpation.

**Figure 2:**
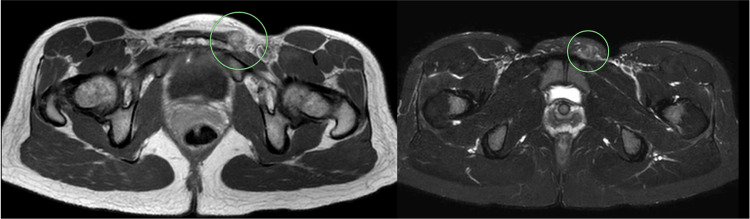
MRI from 16-month follow-up This MRI was performed on the patient approximately 16 months after the original robotic-assisted hernia repair and coincided with her cyclic maximal pain. Note: these images clearly demonstrate inflammation near the left pubic tubercle and the round ligament, which later correlated with endometrial implants on the round ligament. MRI, magnetic resonance imaging.

Given the presence of a palpable, tender mass and the cyclical nature of her symptoms, a repeat MRI was performed, this time scheduled to coincide with the patient’s menstrual cycle. The imaging, shown in Figure 2, revealed soft tissue edema and inflammation in the area of the left inguinal canal along the round ligament near the pubic symphysis. The lesion measured approximately 1.5 cm in diameter. There remained no evidence of hernia recurrence. She was started on oral contraceptive pills as a trial for hormonal symptom suppression, which provided mild relief. Given the suspicion for round ligament endometriosis, she was taken back to the operating room. 

A standard open herniorrhaphy incision was made approximately 2 cm cephalad and parallel to the left inguinal ligament. Upon dissection, the round ligament was found to be inflamed, cystic, and densely adherent to surrounding tissues. It was dissected to its most distal extent and resected near its insertion on the pubic tubercle. An ilioinguinal neurectomy was also performed at that time. The excised round ligament was sent for pathological analysis, which confirmed multiple endometrial implants. Postoperatively, the previously described cyclical pelvic and pubic symphysis pain had resolved, and she remains pain free. She noticed some groin numbness related to her ilioinguinal neurectomy, but this did not cause her any significant distress and improved postoperatively.

## Discussion

Literature review

A brief literature review was performed by searching the PubMed database for medical subject headings (MeSH) terms ("round ligament" OR "inguinal" OR “canal of Nuck”) AND "endometriosis" AND ("hernia" OR "herniorrhaphy" OR "TAPP" OR "TEP" OR "robotic" OR "laparoscopic"). Case reports, comparative studies, evidence synthesis, meta-analysis, observational studies, review, scoping review, and systematic reviews within the last 20 years were included. A total of 35 articles were identified. Non-English language, non-inguinal location, and pediatric data were then excluded. This resulted in 21 articles for inclusion, as detailed in Table 1 below [7-27].

**Table 1:** Clinical characteristics, imaging findings, and management of published cases of inguinal and round ligament endometriosis B, bilateral; CPIP, chronic postoperative inguinal pain; CT, computed tomography; IIN, ilioinguinal neurectomy; L, left; M, mixed laterality; MRI, magnetic resonance imaging; Multi, multiple imaging modalities; NR, not reported; Path, diagnosis established by pathology; R, right; RL, round ligament; US, ultrasonography.

Author	Year	N	Side	Presentation	Imaging	Treatment
Fedele et al. [7]	2007	5	M	Recurrent	US+MRI	RL excision
Ducarme et al. [8]	2007	1	R	Post-repair	US+MRI	Excision + herniorrhaphy
Hagiwara et al. [9]	2007	1	R	Cyclic	CT+MRI	Excision + RL resection
Mashfiqul et al. [10]	2007	1	R	Non-cyclic	US	Wide excision + mesh
Kamio et al. [11]	2009	1	R	Cyclic	US+MRI	Excision
Apostolidis et al. [12]	2009	3	R	Cyclic	US	Excision
Wang et al. [13]	2009	1	L	Cyclic	US+CT	Excision + laparoscopy
Kiyak et al. [14]	2010	1	R	Cyclic	Path	Hernia repair + excision
Jiménez et al. [15]	2011	1	R	Cyclic	CT+MRI	Laparoscopic excision
Prabhu et al. [16]	2013	1	L	Pain/mass	US	Excision
Husain et al. [17]	2015	1	R	Mass	MRI	Excision
Fong et al. [18]	2019	1	R	Mass	None	Excision
Azhar et al. [19]	2019	1	R	Acute	CT+MRI	Hernia repair + excision
AlSinan et al. [20]	2021	1	L	Cyclic	US+CT	Excision + mesh
Chen et al. [21]	2021	1	R	Nodule	US+CT	Excision + herniorrhaphy
Chou et al. [22]	2023	2	M	Pain/mass	US+MRI	Excision ± hormones
Rokhgireh et al. [23]	2024	1	R	Pain	MRI	Laparoscopic excision
Haghgoo et al. [24]	2024	8	M	Swelling	US±MRI	Surgery ± hormones
Lipschultz et al. [25]	2025	1	NR	Pain	MRI	Laparoscopic excision
Takatsuka et al. [26]	2025	6	M	Pain/mass	Multi	Surgical excision
André et al. [27]	2026	1	B	Pain	MRI	Laparoscopic RL excision
Present case	2026	1	L	CPIP	MRI×2	Open RL excision + IIN

Clinical presentation was reported in most studies and was characterized predominantly by groin pain and a palpable inguinal mass. Pain was reported in 18 of 21 (85.7%) studies, while a palpable mass, swelling, nodule, or groin lesion was described in 16 (76.2%) studies. Cyclic or catamenial symptoms were reported in 12 (57.1%) studies, although several reports documented non-cyclic or atypical presentations [23,24].

Right-sided disease predominated throughout the literature. Right-sided involvement was reported in 13 (61.9%) studies, whereas left-sided disease was reported in 4 (19.0%) studies. Bilateral involvement was uncommon and described only by André et al. [27]. Several case series similarly emphasized a strong right-sided predominance, consistent with the historically reported distribution of inguinal endometriosis. The predominance of right-sided disease appears to be one of the most consistent findings across the surveyed literature. However, anatomic involvement varied among reports. Round ligament involvement was specifically described in 11 of 21 (52.4%) studies of the included studies [9,12,16,23,24]. Additional sites of involvement included the canal of Nuck, abdominal wall scar tissue, subcutaneous soft tissues, and other structures within the inguinal canal [8,13,15]. These findings appear generally consistent with the previously described Niitsu classification system [6]. 

Misdiagnosis or delayed diagnosis appears common, with hernias, lymphadenopathy, or hydroceles representing commonly confused pathologies [20,24]. Ultrasonography was the most commonly reported imaging modality and was used in 15 of 21 (71.4%) studies, while MRI was used in 14 of 21 (66.7%) studies, often as an adjunctive modality to further characterize lesions identified on physical examination or ultrasonography. Computed tomography was reported in 7 of 21 (33.3%) studies. Despite advances in imaging, histopathologic examination following surgical excision remained the definitive diagnostic method.

Prior abdominal or pelvic surgery was commonly reported, particularly cesarean delivery and prior gynecologic procedures [8,17,19]. Several studies additionally documented concurrent pelvic endometriosis, infertility, dysmenorrhea, or ovarian endometrioma, suggesting substantial overlap between inguinal disease and more typical manifestations of endometriosis [7,24]. Nevertheless, isolated inguinal disease without known pelvic involvement was also reported in multiple cases [10,16,20].

Management was overwhelmingly surgical. Surgical treatment was reported in all 21 studies providing treatment information (100%), most commonly consisting of complete excision of the lesion with resection of the involved round ligament, hernia sac, or adjacent soft tissue. Reported outcomes were generally favorable, with most studies describing symptom resolution and low rates of recurrence following surgical management.

Case analysis

This case presentation represents a previously undescribed type II inguinal endometriosis following a minimally invasive inguinal hernia repair produced CPIP. Several unique components of this presentation warrant discussion. First, this case represents a relatively rare left-sided presentation of inguinal endometriosis. Interestingly, the patient initially noted right-sided pain before her hernia repair. Her left-sided symptoms, however, were new following her robotic inguinal hernia repair. The relative rarity of left-sided inguinal endometriosis combined with the temporal relationship to her TAPP repair raises the possibility of direct endometrial implant seeding during her operation as a possible pathogenesis. While this is a single case and cannot be construed as causality, surgeons may consider a patient’s previous gynecologic history carefully when evaluating chronic inguinal or CPIP-type symptoms. 

This case also highlights both the difficulty in diagnosing inguinal endometriosis and the need for a rigorous workup during CPIP evaluation. While cyclical symptoms were reported frequently in the literature, this case highlights that imaging findings diagnostic of inguinal endometriosis may also be catamenial. In this case, the patient reported specific symptoms, which were consistent with inguinal endometriosis, and the surgical team held a high clinical suspicion. However, the initial imaging, which was not timed with her symptoms, did not provide any diagnostic utility. This case highlights the importance of timing imaging studies with symptomatic windows to maximize diagnostic value. 

Finally, this case highlights the importance of providing a thorough evaluation for patients with CPIP, as some causes may be reversible. Key clinical insights that this patient’s presentation did not fit typical CPIP included cyclical pain, point tenderness to palpation outside of the expected operative field, and failure of traditional pain management techniques. A generic algorithmic approach to treating these patients without a thorough history and examination may have led to unnecessary morbidity for this patient. The evaluation of CPIP requires a thorough history and examination not only to rule out hernia recurrence but also to evaluate for causes of potentially reversible symptoms, as demonstrated in this case. A multidisciplinary approach offers an opportunity for thoughtful and tailored evaluations and may benefit from earlier gynecologist involvement in cases of atypical CPIP in female patients.

## Conclusions

Round ligament endometriosis is a rare but important cause of groin pain that may mimic CPIP in women of reproductive age. This case demonstrates that cyclical symptoms, atypical pain location, and failure of conventional CPIP therapies should prompt consideration of alternative diagnoses. Key features that suggested the diagnosis were point tenderness and cyclic symptom presentations. Timing imaging studies to coincide with symptomatic periods may improve diagnostic yield for catamenial pathology. Surgical excision remains an effective treatment and can result in complete symptom resolution. 

## References

[1] Sekhon Inderjit Singh HK, Massey LH, Arulampalam T, Motson RW, Pawa N (2022). Chronic groin pain following inguinal hernia repair in the laparoscopic era: systematic review and meta-analysis. Am J Surg.

[2] Chu Z, Zheng B, Yan L (2024). Incidence and predictors of chronic pain after inguinal hernia surgery: a systematic review and meta-analysis. Hernia.

[3] Fitzgibbons RJ Jr, Forse RA (2015). Clinical practice. Groin hernias in adults. N Engl J Med.

[4] (2018). International guidelines for groin hernia management. Hernia.

[5] Sun Z-J, Zhu L, Lang J-H (2010). A rare extrapelvic endometriosis: inguinal endometriosis. J Reprod Med.

[6] Niitsu H, Tsumura H, Kanehiro T, Yamaoka H, Taogoshi H, Murao N (2019). Clinical characteristics and surgical treatment for inguinal endometriosis in young women of reproductive age. Dig Surg.

[7] Fedele L, Bianchi S, Frontino G, Zanconato G, Rubino T (2007). Radical excision of inguinal endometriosis. Obstet Gynecol.

[8] Ducarme G, Uzan M, Poncelet C (2007). Endometriosis mimicking hernia recurrence. Hernia.

[9] Hagiwara Y, Hatori M, Moriya T, Terada Y, Yaegashi N, Ehara S, Kokubun S (2007). Inguinal endometriosis attaching to the round ligament. Australas Radiol.

[10] Mashfiqul MAS, Tan YM, Chintana CW (2007). Endometriosis of the inguinal canal mimicking a hernia. Singapore Med J.

[11] Kamio M, Nagata T, Yamasaki H, Yoshinaga M, Douchi T (2009). Inguinal hernia containing functioning, rudimentary uterine horn and endometriosis. Obstet Gynecol.

[12] Apostolidis S, Michalopoulos A, Papavramidis TS, Papadopoulos VN, Paramythiotis D, Harlaftis N (2009). Inguinal endometriosis: three cases and literature review. South Med J.

[13] Wang CJ, Chao AS, Wang TH, Wu CT, Chao A, Lai CH (2009). Challenge in the management of endometriosis in the canal of Nuck. Fertil Steril.

[14] Kiyak G, Ergul E, Sarikaya SM, Yazgan A (2010). Endometriosis of the groin hernia sac: report of a case and review of the literature. Hernia.

[15] Jiménez JS, Barbero P, Tejerizo A, Guillén C, Strate C (2011). A laparoscopic approach to Nuck's duct endometriosis. Fertil Steril.

[16] Prabhu R, Krishna S, Shenoy R, Thangavelu S (2013). Endometriosis of extra-pelvic round ligament, a diagnostic dilemma for physicians. BMJ Case Rep.

[17] Husain F, Siddiqui ZA, Siddiqui M (2015). A case of endometriosis presenting as an inguinal hernia. BMJ Case Rep.

[18] Fong KN, Lau TW, Mak CC, Lui KW (2019). Inguinal endometriosis: a differential diagnosis of right groin swelling in women of reproductive age. BMJ Case Rep.

[19] Azhar E, Mohammadi SM, Ahmed FM, Waheed A (2019). Extrapelvic endometrioma presenting as acute incarcerated right inguinal hernia in a postpartum patient. BMJ Case Rep.

[20] AlSinan FM, Alsakran AS, Foula MS, Al Omoush TM, Al-Bisher H (2021). Inguinal endometriosis in a nulliparous woman mimicking an inguinal hernia: a case report with literature review. Am J Case Rep.

[21] Chen PC, Cheng CH, Ding DC (2021). Primary inguinal subcutaneous endometriosis accompanied with an inguinal hernia: a case report. Medicine.

[22] Chou CW, Lai PT, Huang CC, Hong JB, Tai YJ (2023). Primary spontaneous inguinal endometriosis: two cases with emphasis on the diagnostic approach. Taiwan J Obstet Gynecol.

[23] Rokhgireh S, MehdizadehKashi A, Noroozi SG, Aminzade Z, Derakhshan R (2024). Laparoscopic surgery for endometriosis of the round ligament: a case of a patient with right-sided inguinal pain. Womens Health (Lond).

[24] Haghgoo A, Faegh A, Mostafavi SR, Zamani HR, Ghahremani M (2024). Inguinal endometriosis: a case series and review of the literature. J Med Case Rep.

[25] Lipschultz RA, Lee TT (2025). Inguinal canal endometriosis. Fertil Steril.

[26] Takatsuka S, Kataoka H, Ito F, Shimura K, Mori T (2025). Pathogenesis, diagnosis, and management of inguinal endometriosis: a case series of six patients. Reprod Sci.

[27] André M, Figuier C, Chauveau B, Canis M (2026). Laparoscopic treatment of bilateral inguinal round ligament endometriosis. J Minim Invasive Gynecol.

